# Data-Driven Metabolic Pathway Compositions Enhance Cancer Survival Prediction

**DOI:** 10.1371/journal.pcbi.1005125

**Published:** 2016-09-27

**Authors:** Noam Auslander, Allon Wagner, Matthew Oberhardt, Eytan Ruppin

**Affiliations:** 1 Center for Bioinformatics and Computational Biology and the Department of Computer Science, University of Maryland, College Park, Maryland, United States of America; 2 Department of Electrical Engineering and Computer Science and the Center for Computational Biology, University of California, Berkeley, Berkeley, California, United States of America; 3 The Blavatnik School of Computer Science and the Sackler School of Medicine, Tel Aviv University, Tel Aviv, Israel; National Center for Biotechnology Information (NCBI), UNITED STATES

## Abstract

Altered cellular metabolism is an important characteristic and driver of cancer. Surprisingly, however, we find here that aggregating individual gene expression using canonical metabolic pathways fails to enhance the classification of noncancerous vs. cancerous tissues and the prediction of cancer patient survival. This supports the notion that metabolic alterations in cancer rewire cellular metabolism through unconventional pathways. Here we present MCF (Metabolic classifier and feature generator), which incorporates gene expression measurements into a human metabolic network to infer new cancer-mediated pathway compositions that enhance cancer vs. adjacent noncancerous tissue classification across five different cancer types. MCF outperforms standard classifiers based on individual gene expression and on canonical human curated metabolic pathways. It successfully builds robust classifiers integrating different datasets of the same cancer type. Reassuringly, the MCF pathways identified lead to metabolites known to be associated with the pertaining specific cancer types. Aggregating gene expression through MCF pathways leads to markedly better predictions of breast cancer patients’ survival in an independent cohort than using the canonical human metabolic pathways (C-index = 0.69 vs. 0.52, respectively). Notably, the survival predictive power of individual MCF pathways strongly correlates with their power in predicting cancer vs. noncancerous samples. The more predictive composite pathways identified via MCF are hence more likely to capture key metabolic alterations occurring in cancer than the canonical pathways characterizing healthy human metabolism.

## Author Summary

Cancer proliferating cells adapt their metabolism to support the conversion of available nutrients into biomass, which often involves an increased rate of specific metabolic pathways, such as glycolysis. Surprisingly, however, we observe that aggregating individual gene expression using canonical human metabolic pathways frequently fails to enhance the classification of noncancerous vs. cancerous tissues and in the task of predicting cancer patient survival. This supports the notion that metabolic alterations in cancer rewire cellular metabolism through unconventional pathways. Here we introduce a novel algorithm (MCF) that aims to identify these cancer-mediated ‘composite’ metabolic pathways by identifying those that best differentiate between cancerous vs. non-cancerous tissues gene expression. Remarkably, MCF successfully builds robust classifiers integrating different datasets of the same cancer type. We further show that the data-driven pathways identified by MCF, in contrast to the canonical literature-based pathways, successfully generate clinically relevant features that are predictive of breast cancer patients’ survival in an independent dataset. Our findings thus suggest that cancer metabolism may be rewired via non-standard composite pathways.

## Introduction

In recent years the study of cancer metabolism gained renewed interest as means to understand cancer’s emergence, pathophysiology, and for finding candidate targets for therapeutics [1–6]. Metabolism is universally conceptualized through the abstraction of *pathways*, which are groups of enzymatic reactions thought to operate coherently [7]. Undoubtedly, this abstraction is very useful and underlies many studies [8]. In cancer, Hu et al. [9] showed that changes in the aggregate expression of canonical metabolic pathways that occur in individual tumors are reproducible in independent samples of the same tumor. On the other hand, it has also been observed that the canonical pathways abstraction does not capture the complexity of the metabolic network in full; Bordbar et al. [10] recently presented an algorithm for deriving metabolic pathways based on the principle of parsimonious use of cellular components. They showed that it produces pathways that are more biologically plausible than the human defined ‘canonical’ pathways present in databases such as KEGG, EcoCyc, YeastCyc, and Gene Ontology. Moreover, cancer cells drastically alter their metabolic functions [11,12] and as a result the canonical metabolic pathways, which have been historically constructed to characterize healthy metabolism, may not suit them as much.

Here we turn to study whether the expression of metabolic pathways as a whole is predictive of cancer’s outcome and prognosis. One may expect that pooling information across genes in a pathway would be an effective method for feature generation by mitigating experimental noise in the measurement of individual genes. There has been therefore considerable interest in cancer classifiers that utilize network- and pathway-based meta-features [13–16] However, recent studies reported that many of these classifiers do not outperform models trained over single gene features [17–19]. Similarly, we find here that grouping gene expression by canonical metabolic pathways fails to enhance the prediction of patient survival and prognosis across ten datasets of five cancer types. This goes along with the intuition that cancer rewires cellular metabolism in a way that gives rise to non-standard pathways, which are probably unique to each tumor type. To address this, we introduce a novel algorithm (MCF) that aims to identify unsupervised cancer-mediated functional metabolic pathways from the tumor’s (vs. non-cancerous) gene expression. By limiting the problem space to the metabolic subsystem, we reduce dimensionality and simplify the learning task, while preserving essential information, as metabolism is known to be pivotal in tumor growth and proliferation. Furthermore, the metabolic network is highly structured and has been intensively characterized. Most of its components have been manually curated and supported by direct experimental evidence [20], possibly going beyond other networks (e.g., PPI) that have been inferred from high-throughput biological experiments that could thus contain higher levels of noise. We show that the data-driven pathways identified by MCF, in contrast to the canonical literature-based pathways, successfully generate clinically relevant features that are predictive of patients’ survival.

## Results

### The Metabolic classifier and feature generator (MCF) algorithm

We first tested if the use of canonical pathways enhances the accuracy of cancer classification. We overlaid gene expression data derived from 3611 samples across ten datasets of five cancer types (including breast, lung, colon, prostate and head and neck squamous cell carcinoma) onto canonical metabolic pathways defined in the RECON1 human metabolic model [20] and quantified the expression of every metabolic pathway based on the sum of the expression of all genes associated with this pathway (Methods, which in this case yields better performance than using the mean expression). We then trained SVM classifiers of cancer vs. adjacent noncancerous tissue samples using either the expression of individual metabolic genes (henceforth, MGE-SVMs) or human canonical metabolic pathways’ expression (Methods). Testing the classifiers in five-fold cross validation we found that using the canonical pathway expression leads to inferior performance in these classification tasks compared to using the individual metabolic gene expression (S1 Fig). These findings motivated us to identify pathways whose activity may better reflect the altered rewiring of metabolism in cancer and enhance cancer prediction.

**Fig 1:**
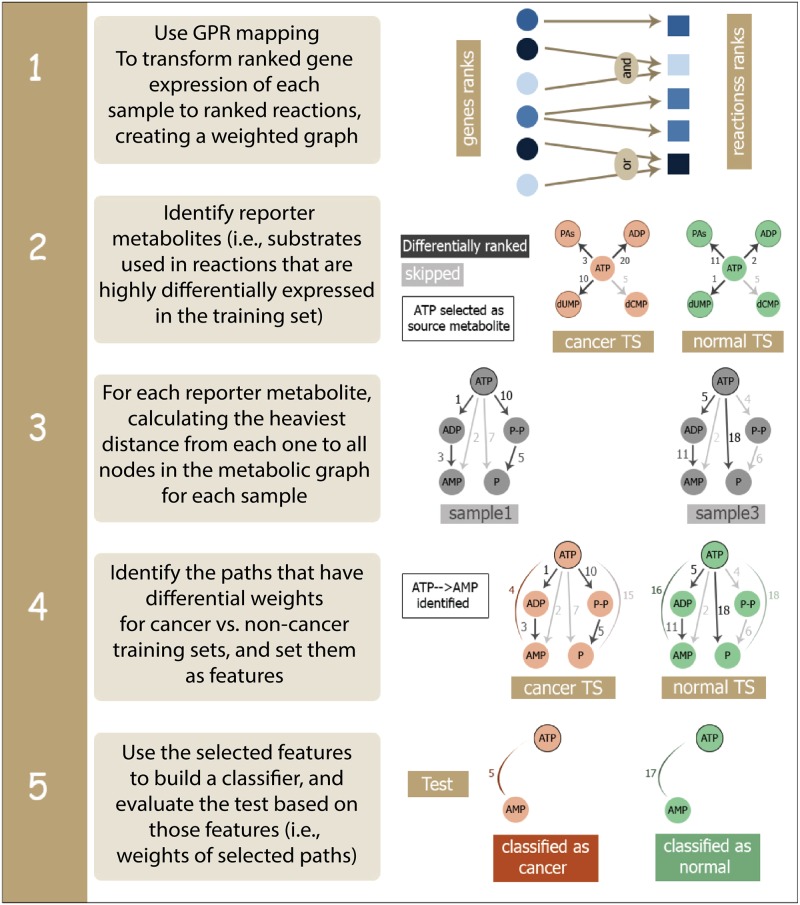
Overview of the MCF algorithm.

To this end we developed a new data-driven algorithm, called the Metabolic classifier and feature generator (MCF): (1) We first define a differentially expressed reaction as a reaction whose ranked expression level within a sample is significantly different in noncancerous vs. cancerous samples (using a Wilcoxon rank-sum p-value with *α* = 0.05, Methods). (2) The next step of MCF follows the concept of *reporter metabolites* [21]—it identifies metabolites that participate in differentially expressed reactions between the noncancerous and cancerous samples. (3–4) The key novelty of MCF is to use these reporter metabolites as centerpieces for building novel *composite pathways* leading from each reporter metabolite s to a group of target metabolites T_s_ that show consistent differential expression between the cancerous and noncancerous states. These pathways are (by construction) predictive of the cancer vs. non-cancer states. (5) We then build a support vector machine (SVM-MCF) ensemble classifier of cancer vs. noncancerous tissue based on the gene expression of the new composite pathways as classification features. We apply a five-fold cross validation procedure to test the classification rate (accuracy) and area under the cover (AUC) for each dataset studied (Methods). The main steps of MCF are outlined below and in Fig 1 (see Methods for a formal description):

1. **Rank-transform the gene expression data:** We first rank-transform the gene expression data and convert it biochemical reaction expression values using the human model’s genes-to-reactions mapping. This results in patient specific weighted metabolic networks in which the weights of each reaction edge correspond to the rank assigned to this reaction for a certain patient.
2. **Identify seed reporter metabolites:** For computational tractability, we limited the search to simple paths in which the first reaction is differentially expressed between the two states. To this end, we identify metabolites that are substrates in a large number of reactions that are differentially expressed between cancerous and noncancerous samples.
3. **Assigning ‘expression weights’ from each seed reporter metabolite on the paths to all other metabolites in the network:** We calculate the heaviest distances (i.e. the weight of a simple path with the largest sum of reactions’ expression values) from each seed metabolite to all other metabolites in the network. For the purpose of identifying the new composite paths, the metabolic network hypergraph is transformed to a regular graph representation having metabolite nodes and (directed) edge connecting any two metabolites that participate in a given reaction as a substrate and a product, respectively (if the reaction is directed).
4. **Identify the most differentially expressed (‘heaviest’) pathways:** For each source metabolite *s* we find the L = 10 target metabolites T_s_ such that the heaviest distance from *s* leading to each of the targets in T_s_ differs most between the noncancerous and cancer training sets.
5. **Building an SVM classifier:** For each of the N source metabolites *s* we train an independent SVM classifier to distinguish cancerous from noncancerous samples using the weight of the L selected paths from *s* to T_s_ as features. This results in an ensemble of N SVMs. A test sample is then classified by a majority vote over the N classifiers.

### The predictive performance of MCF in classifying cancerous vs noncancerous tissues and associated biomarkers

We compared the accuracy of the MCF to MGE-SVMs classifiers that are based on individual metabolic gene expression by comparing their AUC and mean accuracy scores in a five-fold cross validation on various cancerous vs noncancerous classification tasks. We find that MCF performs as well as MGE-SVM in all 10 datasets studied spanning five different cancer types, and significantly outperforms MGE-SVM in five of these datasets (S2 Fig; S1 Table).

**Fig 2:**
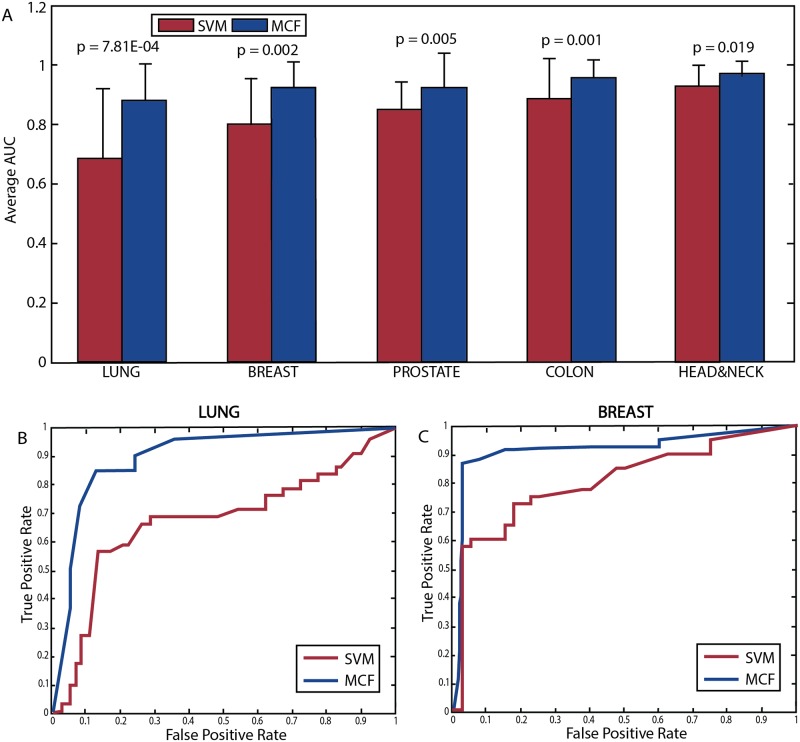
Comparing the performance of MCF to MGE-SVM across integrated cancer-type datasets. (A) A bar plot describing the predicted AUC obtained over the combined datasets of the same cancer type using a five-fold cross validation procedure for MGE-SVM (red bars) and MCF (blue bars) classifiers. AUC denotes the area under the curve. Error bars represent one standard deviation, and p-values are for a one-sided, paired-sample t-test for the AUC of each of the five folds. (B), (C) present the receiver operating characteristic (ROC) curves obtained in the classification of the lung and breast cancer combined datasets, respectively.

As MCF aggregates transcriptional information in network-based manner, we hypothesized that it will be more robust than MGE-SVM when trained on data of the same cancer type but aggregated from multiple studies. To test this we merged the available tumor/tissue samples expression (rank-transformed, Methods) data from both GEO and TCGA, producing a combined dataset for each of the five different cancer types studied. We compared the performance (AUC and accuracy) of MCF and MGE-SVM on each of the five combined datasets using a standard five-fold cross-validation procedure. Combining datasets in this manner accentuated the higher predictive performance of MCF vs. MGE-SVM across all cancer types studied (Fig 2, S2 Table), including colon cancer where no significant performance difference was observed previously (S2 Table).

Notably, source metabolites that strongly differ in usage between noncancerous and cancerous tissues may constitute interesting cancer biomarker candidates. We find that there is a small set of such source metabolites that recur in multiple cancer types (see S3 Table), and they vanish in randomly shuffled data (S4 Table). These include currency energy metabolites (e.g., NAD+ and ATP), a finding consistent with the large alterations seen in energy metabolism in cancer. We examined the target metabolites T_s_ that contribute most to ATP being differentially utilized. As the paths leading to them from ATP are most differentially expressed, this may testify that the consumption of ATP to produce each of these metabolites is altered in cancer (and may possibly serve as correlate to their overall production levels). These target metabolites are specific for cancer type (Table 1, a pattern that remained robust to the introduction of noise to the data (See Methods and S5 Table)). This suggests that while ATP is differentially utilized between tumors and their noncancerous tissues counterparts in all cancer types, there exists considerable variance in the ways it is utilized.

**Table 1:** The target T_s_ metabolites that MCF selected when it choses ATP as a seed (↑ denotes increased formation from ATP in cancer and ↓ denotes decreased formation from ATP in cancer compared to noncancerous tissue counterpart, Methods). The table shows one instance of each selected target although in some cases the same target metabolite was identified in multiple compartments (e.g. UDP in the cytosol and in the mitochondria).

prostate	Breast	Colon	head & neck	lung
↑ 3alpha,7alpha,12alpha-Trihydroxy-5beta-cholestanoyl-CoA(S)	↑ dADP	↓ O-Acetylcarnitine	↑ CTP	↑ Hydroxy-methylglutaryl-CoA
↑ 3alpha,7alpha-Dihydroxy-5beta-cholest-24-enoyl-CoA	↑ Oxidized thioredoxin	↑ 5-Phospho-beta-D-ribosylamine	↑ dATP	↑ Spermine
↓ 3alpha,7alpha,26-Trihydroxy-5beta-cholestane	↓ Hydrogen peroxide	↑ Spermine	↑ dCTP	↑ D-Mannose 1-phosphate
↓ 3alpha,7alpha,12alpha-Trihydroxy-5beta-cholestan-26-al	↓ L-Threonate	↑ Fumarate	↑ dGTP	↑ Deoxycytidine
↓ 7alpha-Dihydroxy-5beta-cholestan-26-al	↓ Hydrogen peroxide	↑ GMP	↑ dITP	↑ Diphosphate
↓ 3alpha,7alpha,12alpha,26-Tetrahydroxy-5beta-cholestane	↓ Iodine	↓ retinoyl glucuronide	↑ dTTP	↑ UDP-D-glucuronate
↑ 5-Amino-1-(5-Phospho-D-ribosyl)imidazole-4-carboxamide		↓ UDP		↑ Phosphoenolpyruvate
		↑ Leukotriene B4		↓ Oxalate

Several of the target metabolites are known to be associated with their respective cancers: Oxalate has been studied as a survival marker in lung cancer [22]; spermine has been observed to be differentially expressed in lung and colon cancer [23–25]; Carnitine was shown to slow down tumor development in colon cancer [26]; and blockage of Leukotriene B4 was reported to suppress cell proliferation in colon cancer patients [27]. Thus, MCF identifies key metabolites that take part in metabolic processes that are altered in the specific cancers they occur.

### MCF prediction of patients’ survival

As we have shown, MCF generates new composite pathways that show more power than traditional pathways in classifying normal versus cancer samples. To evaluate the clinical significance of these new features we examined whether they are also predictive of a different objective, the prediction of survival of breast cancer patients. Furthermore, to test whether the clinical utility of MCF pathways carried between datasets, we trained and tested the pathways on *independent* datasets. For training we used the combined GEO and TCGA breast cancer data. For testing, we used an independent dataset (METABRIC, [28]) that includes gene expression measurements from 1,981 cancer patients and their corresponding survival information. Remarkably, we find that out of the 80 pathways that MCF identified as differentially expressed in the original classification task on the combined TCGA and GEO data (L = 10 targets from 8 identified source metabolites, see S3 Table), 58 pathways are predictive for survival in the METABRIC data using Kaplan-Meier estimator [29] (FDR corrected Kaplan-Meier log-rank p-value < 0.05; methods). In marked contrast, the expression levels of *none* of the canonical metabolic pathways defined by Recon1 are predictive of survival in this dataset (S3 Fig). This is in line with our previous observation that the activity of the canonical metabolic pathways is not helpful in distinguishing between cancerous vs. noncancerous samples.

**Fig 3:**
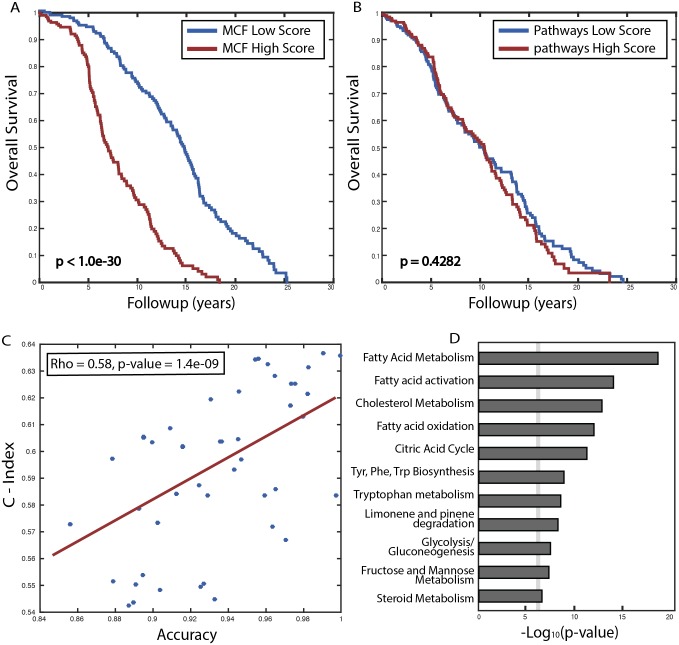
MCF pathway utilization predicts the survival of breast cancer patients, while canonical pathways show no such signal. Shown in (A) and (B) are the Kaplan-Meier survival curves for patients predicted by MCF and canonical pathways respectively to have the best and worst prognosis (top and bottom 10% of patients scores, respectively; Methods). (C) A scatter plot showing the correlation between the prediction classification accuracy achieved using each individual MCF pathway in the combined breast cancer data from TCGA and GEO (where they are identified) (X-label) and the C-index obtained using each such pathway in predicting patients’ survival on the (unseen) METABRIC data. (D) The canonical pathway enrichment of the reactions participating in the MCF composite pathways predictive of survival. The dashed line represents a significance threshold of 0.05 (corrected for multiple hypotheses testing).

To evaluate the aggregate predictive power of the set of pathways selected by MCF as a whole, we compared patients predicted by MCF to have the best and worst prognosis (top and bottom 10%, respectively; Methods) and found that they indeed have a marked difference in their survival as predicted (Fig 3A, delta-AUC = 0.2436, and Kaplan-Meier log-rank P-value < 1.0e-30). In contrast, when aggregating information across the canonical human metabolic model pathways in a similar manner we find that pathways predicted to have best and worst prognosis show no difference in survival (Fig 3B, delta-AUC = 0.0176, and Kaplan-Meier log-rank P-value = 0.4282). We then examined whether the aggregated pathway score can be used as a survival model for the METABRIC dataset, using the conventional concordance index (C-index) [30]. We find that while the pathways selected by MCF are predictive of patients survival, the canonical human metabolic model pathways do not show such predictive power (C-index = 0.69 vs. 0.52, respectively). Interestingly we find that the predictive power of individual MCF selected pathways in the original task of predicting cancer vs. noncancerous samples (i.e. the AUC obtained from the cross validation procedure on the combined datasets from TCGA and GEO) markedly correlates with their predictive power for survival in the METABRIC dataset (Spearman *ρ* = 0.58, p-value <1.4e-09). This finding explains their predictive power across these different tasks and datasets, and further testifies to their clinical significance.

Finally, we performed a canonical pathway enrichment analysis over the reactions participating in the MCF composite pathways identified in breast cancer that are predictive of survival. We find that the most enriched canonical pathways emerging in this analysis are already known to be associated with cancer initiation and progression, such as fatty acid related metabolic pathways [31–33], the citric acid cycle [34,35] and cholesterol and steroid metabolism [36] (Fig 3D). Hence, even though aggregated gene expression through canonical pathways does not show survival predictive power, the composite alterations in cancer do rewire its metabolism using components of these traditional pathways, albeit via different composition.

## Discussion

We present a novel method termed MCF that identifies data-driven pathway compositions that best differentiate the metabolic alterations occurring in cancerous vs. noncancerous tissues. MCF leverages a priori knowledge on the structure of the human metabolic network (ignoring its conventional decomposition to canonical pathways) to inform the analysis of cancer vs. noncancerous gene expression. It detects key hubs of metabolic alterations and infers the composition of non-standard pathways altered in a specific cancer type. Applied across five different cancer types, we find that MCF outperforms standard methods in the basic task of cancer vs. noncancerous classification. Remarkably, MCF derived pathways successfully predict patients’ survival in an independent dataset while standard metabolic pathways fail to do so, testifying on the robustness and utility of the metabolic features learned by MCF.

Meta-learning is of great relevance to cancer classification as it can potentially exploit one of the hallmarks of cancer, deregulation of pathways and cellular processes, by taking knowledge on relations between genes and pathways into account in the classifier [16,37,38]. However, recent studies have reported that many of these methods do not outperform a model trained over single gene features [17–19,39]. MCF offers a solution to some of the main issues that hampered previous methods. First, some previous studies are based on pre-defined gene sets [40] or networks [41] characterizing healthy cells while cancer may rewire many functions, and in particular its metabolism. To this end, MCF performs unsupervised pathway generation and selection that captures key metabolic alterations occurring in cancer. Second, some studies relied on the topology of a pre-defined biological network such as a co-expression network [41], cellular pathway map [42] or protein–protein interaction (PPI) network [43] that have been inferred from high-throughput studies. In difference, MCF relies on a manually curated metabolic network that is extensively supported by experimental evidence [20]. The metabolic network is thus less noisy, while still highly informative due to metabolism’s role in cancer growth and development. Third, it has been shown that structural and directional information improves the predictive power of meta-features over single genes [39]; In accord, the metabolic network is directional and highly structured which allows MCF to infer pathways of biological relevance.

While metabolic reprogramming is a substantial part of cancer biology, the methodological insights obtained from developing MCF are general, and could potentially be built into path-centric approaches that would involve other cellular networks. This could lead to stronger predictors based on reliable models of signaling and regulatory networks on a genome scale. Second, finding the most separating paths in differently weighted graphs is an NP-complete problem. Here, we only offer a heuristic solution that is obviously sub-optimal. This could be improved upon by employing more exhaustive and/or efficient weighted path searching methods. We can expect that follow-up work will advance the identification of top separating pathways in differentially weighted metabolic graphs, potentially improving the power of MCF further.

In summary, we show that integrating gene expression measurements within a genome-scale map of human metabolism via MCF results in the identification of clinically relevant features capable of predicting survival, while enhancing cancer classification power from gene expression data. We believe that future applications of MCF may help identify cancer specific onco-metabolites and advance our understanding of metabolic alterations in cancer.

## Materials and Methods

### Gene expression datasets

We focused on five cancer types, and for each one utilized datasets from TCGA [44] and GEO [45], as summarized in Table 2.

**Table 2:** summary of the datasets utilized in this work for five cancer types. N and C stand for number of normal and cancerous samples in the data, respectively.

	TCGA data		GEO data	
Cancer type	TCGA designation	sample count (N/C)	GEO accession	sample count (N/C)
Prostate	PRAD	487/52	GSE32448 [46]	40/40
Lung adeno-carcinoma	LUAD	58/490	GSE19804 [24]	60/60
Colon	COAD	41/273	GSE32323 [47]	17/17
Head & neck	HNSC	43/498	GSE6631 [48]	22/22
Breast	BRCA	111/1098	GSE10780 [49]	140/42

In addition, we used the METABRIC breast cancer database by Curtis et al. [28] to test the predictive power of MCF pathways with respect to patient survival.

### Evaluation of classifiers

Throughout this study, we evaluate classifier performance by computing the AUC and average accuracy in a five-fold cross-validation procedure. We repeated 100 times the following:

- Down-sample either the cancerous or normal groups: Assume that the data has N normal samples and C cancerous samples and |N|>|C|. We randomly chose |C| samples out of the normal group and excluded the rest. Similarly, if the data had more cancerous samples than normal ones, we down-sampled the cancerous group to the size of the normal group. This ensures that the accuracy statistic is not biased due to an over-representation of one of the groups, which occurs in many of the datasets studied here.
- 5-fold cross validation: We split the chosen samples into 5 folds, each time training on 4/5 of them and testing by computing the AUC or accuracy on the remaining 1/5.

The AUC and accuracy shown here is the average of the 100 repetitions, and the paired t-test p-values are from the resulting vector of 100 AUC or accuracy values for each such random selection.

### Metabolic gene expression SVMs (MGE-SVMs)

To classify cancer vs. normal samples according to metabolic gene expression, we trained a support vector machine (SVM) using the expression of 1,496 metabolic genes as features. We denote these machines MGE-SVMs. Metabolic genes are defined in this study as the set of 1,496 genes annotated in Recon1 [20] a well-curated reconstruction of the global human metabolic network.

We observed that SVMs trained on this reduced set of gene expression features consistently outperformed SVMs trained on the expression of all genes. This is not surprising seeing that the metabolic subset has roughly one-order of magnitude smaller dimensionality, and yet remains highly informative because of the key role of metabolic adaptations in cancer [1,50,51]. Applying further dimensionality reduction on the set of 1,496 metabolic genes (e.g., through PCA) had little effect on the results. In addition, we observed that training MGE-SVMs with ranked expression values (that we use for MCF) achieves similar, but slightly inferior, results to the ones obtained using the expression values themselves.

### Converting gene expression into biochemical reaction expression

Recon1 defines a mechanistic genotype-phenotype relationship through Boolean rules that encode gene-protein-reaction (GPR) associations. To convert ranked gene expression to biochemical reaction expression, we evaluated the Boolean GPR rule of that reaction while replacing the “AND” and “OR” operators with “min” and “max”, respectively as described in [52]. Differential expression between biochemical reaction is determined by a Wilcoxon rank sum test with a significance threshold of 0.05, Bonferroni-adjusted for multiple hypotheses where appropriate.

### Computing metabolic pathway expression

Classification based on metabolic pathways relied on the pathway definitions embedded in Recon1, which associates every reaction with a single pathway out of a total of 99 pathways defined based on the Kyoto Encyclopedia of Genes and Genomes (KEGG) LIGAND database. To compute a pathway expression, we first converted the ranked gene expression to ranked reaction expression as described above, and then summed the ranked expression of all the reactions associated with the pathway. An alternative methods of computing pathway in which for each pathway we use the sum the ranks of all its associated genes showed inferior performance comparing to the method presented here, as well as using the mean of ranked reaction expression instead of the sum.

### Identifying seed reporter metabolites

MCF builds metabolic pathways that have highly differential expression between the two target states (i.e., cancerous and non-cancerous). However, identifying the most differentially expressed pathways between two groups of weighted networks is a NP-hard problem by reduction from the problem of finding the longest-path [53] (Given a directed weighted graph G, let w be the smallest weight in G. Create a copy G’ of G with all edge weights set to w-c for some constant c>0. The most differentiating path between G and G’ is the heaviest (i.e., longest) path in G). For computational tractability, we limited the search for simple paths in which the first reaction is differentially expressed between the two states. We chose source metabolites that are substrates in at least k> = 5 differentially expressed reactions with Wilcoxon rank-sum p-value corrected for multiple hypothesis.

### Building the classifier

To build a classifier based on the differential expression of the pathway from source metabolite *s* to L = 10 target metabolites, we do the following: we compute the heaviest distances (i.e. the weight of a simple path with largest sum of reactions expression values) from *s* to the all other metabolites in the network in all of the train samples. For the purpose of computing paths, we followed the common approach [54,55] transforming the hypergraph into a digraph and limiting ourselves to pathways that are simple directed paths in the digraph. The metabolic hypergraph is viewed as a standard graph with metabolite nodes and a directed edge (u,v) connecting any two metabolites such that u and v participate in some reaction as a substrate and a product, respectively. We then select a set T_s_ of L target metabolites for which the paths from *s* were most differentially expressed. I.e., for every target metabolite *t* we compute the Wilcoxon rank sum p-value when comparing the heaviest distance from *s* to *t* in the normal vs. the cancer samples, and we finally choose the T_s_ with L metabolites that obtained the smallest p-values out of all possible targets. The distances from *s* to the chosen L metabolites (denoted T_s_) are used as features for an SVM.

Let N be the number of source metabolites detected. MCF repeats the procedure described above for each of the source metabolites *s*, and for each *s* a distinct SVM is trained. This results in an ensemble of N SVMs. A test sample is then classified by the majority vote of the N individual classifiers (no ties ever occurred in the present study).

### MCF classification score

The MCF classifier is an ensemble of N SVMs (for each detected source metabolite). The MCF classification score for classifying observation *x* is the sum of N scores assigned to *x* by the N SVMs. Therefore:

$$MCF_{score\left(x\right)}=\sum_{i=1}^{N}f_{i}\left(x\right)$$

Where *f*_*i*_ (*x*) is the predicted response of *x* for the trained classification function *f*_*i*_ (trained on the features selected for source metabolite *i*)

$$f_{i}\left(x\right)=\;\sum_{j=1}^{n}\alpha_{i,j}y_{i,j}G\left(X_{i},X\right)+b_{i}$$

Where (*α*_*i*,1_…*α*_*i*,*n*_, *b*_*i*_) are the estimated parameters, *G*(*X*_*i*_, *X*) is the dot product in the predictor space between *X* and the support vectors and the sum indicates training set observations.

### Predicting patient survival by canonical or MCF pathways

To train the model and select the features we use the combined GEO and TCGA breast cancer datasets and train it on the original classification task of separating noncancerous from cancer tissues (when all samples are used). This results in 80 composite pathways that are generated and selected by MCF (for comparison, the human metabolic network defines 99 different pathways). We then use the METABRIC dataset and calculate the weights of the 80 selected pathways for this dataset (by generating a weighted metabolic graph for each sample in the MTABRIC dataset and calculating the heaviest distance between each seed metabolite and the target metabolites selected for it for the combined dataset from GEO and TCGA) as well as the weight of the 99 human metabolic network pathways. In the two pathways sets, we define the weight of each patient for every pathway by the sum of ranks of the reactions associated with the pathway. For every pathway we evaluated the KM log-rank p-value taking top 10% and bottom 10% weighted pathways.

To calculate an aggregated pathway score using either the 80 MCF selected pathways or the 99 canonical model pathways we calculate the weights of these pathways using the METABRIC gene expression data. We compute for each patient’s tumor two aggregate scores (one over the MCF pathways and over the model pathways) as follows:

$$score\left(patient_{i}\right)=\frac{\sum_{p\in P_{c}}weight_{i}\left(p\right)}{\sum_{p\in P_{n}}weight_{i}\left(p\right)}$$

When *weight*_*i*_(*p*) is the weight of pathway *p* for patient *i*. *P*_*c*_ is the set of pathways (either MCF selected pathways of canonical pathways) in which high expression levels were associated with cancer state, and *P*_*n*_ is the set of pathways in which high expression levels were associated with noncancerous healthy state. Both *P*_*c*_ and *P*_*n*_ are determined by analyzing the two breast cancer datasets from TCGA and GEO (the mean of each pathway was evaluated for noncancerous and cancer samples to decide whether a pathway is in *P*_*c*_ or in *P*_*n*_). These *P*_*c*_ and *P*_*n*_ set of pathways were then used to predict the patients survival an independent METABRIC breast cancer dataset, by assessing *weight*_*i*_(*p*) for every sample based on its transcriptomics and computing *score*(*patient*_*i*_) accordingly. A KM analysis is then employed to examine the survival difference of high score versus low score patients’ samples.

### MCF robustness to gene expression noise

To test MCF’s robustness, we introduced noise into every sample’s gene expression vector by adding random Gaussian noise with distributions N(0,1), N(0,2) and N(0,3). We then trained MCF classifiers based on the perturbed data and evaluated the source and target metabolites MCF selected.

## Supporting Information

S1 Fig A bar plot describing the average accuracy over a five-fold cross validation procedure for MGE-SVM classifiers of cancerous vs. noncancerous samples trained on either individual metabolic gene expression levels (black bars) or aggregate metabolic pathway expression levels (grey bars). Metabolic pathway expression is defined based on the mean of the expression of all genes associated with a pathway (methods). Accuracy denotes the percentage of correctly classified samples (accuracy is an appropriate metric because all datasets had comparable ratios of positive and negative samples, see Methods). Error bars represent one standard deviation, and p-values are for a one-sided, paired-sample t-test for the accuracy of each of the five folds. (G) Stands for datasets from GEO and (T) for datasets from TCGA (refer to Methods table 5.1 for details concerning the datasets studied here).
(DOCX)

S2 Fig (A) A bar plot describing the accuracy over a five-fold cross validation procedure for MGE-SVM classifiers trained on metabolic gene expression levels (red bars) and for MCF classifier (blue bars). Accuracy is the percentage of correctly classified samples. (G) Stands for datasets from GEO and (T) for datasets from TCGA (as in the legend of fig 1, methods). Error bars represent one standard deviation, and p-values are for a one-sided, paired-sample t-test for the accuracy of each of the five folds. (B) Boxplots showing the distribution for the true positive and true negative rates for MGE-SVM and MCF for all 10 datasets evaluated in this study. (C) A receiver operating characteristic (ROC) curve for classification of the GSE32448 prostate dataset, for which the MGE-SVM performance was particularly poor.
(DOCX)

S3 Fig The Kaplan-Meier survival curves of five metabolic pathways that are known to be altered in cancer (as cited in the main text), for patients predicted by these pathways to have the best and worst prognosis (top and bottom 10% of patients scores, respectively). FDR correction with *α* = 0.05 yields threshold of p < 0.0039.
(DOCX)

S1 Table The AUC and average accuracy for SVM and MCF classifiers for each dataset and the accuracy corresponding paired sample t-test p-value for a 5-fold cross validation procedure. (DOCX)

S2 Table The AUC and accuracy for the combined datasets of when using SVM vs. MCF for each cancer type and the accuracy corresponding paired sampled p-values for t-test of the 5-fold cross validation. (DOCX)

S3 Table The source metabolites selected for each cancer type. (DOCX)

S4 Table The frequency of which each breast cancer identified source metabolite was selected over 10,000 repetitions of randomly labeling the datasets and identifying source metabolites that are differentially consumed between cancer and control. (DOCX)

S5 Table The Spearman correlation coefficient (RHO) of (1) the vector of p-values of differential distances vectors from ATP to all other metabolites between cancer and control for the original gene expression datasets and (2) The same vector of p-values for the noisy gene expression datasets for increasing variances. (DOCX)
