# Supplementary material for: Data-Driven Metabolic Pathway Compositions Enhance Cancer Survival Prediction
Source: PLoS Comput Biol. 2016 Sep 27;12(9):e1005125. doi: 10.1371/journal.pcbi.1005125 (PMC5038951; doi:10.1371/journal.pcbi.1005125)
Supplement: S1 Fig — Metabolic pathway expression is defined based on the mean of the expression of all genes associated with a pathway (methods). Accuracy denotes the percentage of correctly classified samples (accuracy is an appropriate metric because all datasets had comparable ratios of positive and negative samples, see Methods). Error bars represent one standard deviation, and p-values are for a one-sided, paired-sample t-test for the accuracy of each of the five folds. (G) Stands for datasets from GEO and (T) for datasets from TCGA (refer to Methods table 5.1 for details concerning the datasets studied here). (DOCX) [file pcbi.1005125.s001.docx]

**S1 Figure** – A bar plot describing the average accuracy over a five-fold cross validation procedure for MGE-SVM classifiers of cancerous vs. noncancerous samples trained on either individual metabolic gene expression levels (black bars) or aggregate metabolic pathway expression levels (grey bars). Metabolic pathway expression is defined based on the mean of the expression of all genes associated with a pathway (methods). Accuracy denotes the percentage of correctly classified samples (accuracy is an appropriate metric because all datasets had comparable ratios of positive and negative samples, see Methods). Error bars represent one standard deviation, and p-values are for a one-sided, paired-sample t-test for the accuracy of each of the five folds. (G) Stands for datasets from GEO and (T) for datasets from TCGA (refer to Methods table 5.1 for details concerning the datasets studied here).
