# Supplementary material for: Data-Driven Metabolic Pathway Compositions Enhance Cancer Survival Prediction
Source: PLoS Comput Biol. 2016 Sep 27;12(9):e1005125. doi: 10.1371/journal.pcbi.1005125 (PMC5038951; doi:10.1371/journal.pcbi.1005125)
Supplement: S2 Fig — (A) A bar plot describing the accuracy over a five-fold cross validation procedure for MGE-SVM classifiers trained on metabolic gene expression levels (red bars) and for MCF classifier (blue bars). Accuracy is the percentage of correctly classified samples. (G) Stands for datasets from GEO and (T) for datasets from TCGA (as in the legend of fig 1, methods). Error bars represent one standard deviation, and p-values are for a one-sided, paired-sample t-test for the accuracy of each of the five folds. (B) Boxplots showing the distribution for the true positive and true negative rates for MGE-SVM and MCF for all 10 datasets evaluated in this study. (C) A receiver operating characteristic (ROC) curve for classification of the GSE32448 prostate dataset, for which the MGE-SVM performance was particularly poor. (DOCX) [file pcbi.1005125.s002.docx]

**S2 Figure** - (A) A bar plot describing the accuracy over a five-fold cross validation procedure for MGE-SVM classifiers trained on metabolic gene expression levels (red bars) and for MCF classifier (blue bars). Accuracy is the percentage of correctly classified samples. (G) Stands for datasets from GEO and (T) for datasets from TCGA (as in the legend of figure 1, methods). Error bars represent one standard deviation, and p-values are for a one-sided, paired-sample t-test for the accuracy of each of the five folds. (B) Boxplots showing the distribution for the true positive and true negative rates for MGE-SVM and MCF for all 10 datasets evaluated in this study. (C) A receiver operating characteristic (ROC) curve for classification of the GSE32448 prostate dataset, for which the MGE-SVM performance was particularly poor.
