# Supplementary material for: Data-Driven Metabolic Pathway Compositions Enhance Cancer Survival Prediction
Source: PLoS Comput Biol. 2016 Sep 27;12(9):e1005125. doi: 10.1371/journal.pcbi.1005125 (PMC5038951; doi:10.1371/journal.pcbi.1005125)
Supplement: S3 Fig — FDR correction with α = 0.05 yields threshold of p < 0.0039. (DOCX) [file pcbi.1005125.s003.docx]

**S3 Figure** – The Kaplan-Meier survival curves of five metabolic pathways that are known to be altered in cancer (as cited in the main text), for patients predicted by these pathways to have the best and worst prognosis (top and bottom 10% of patients scores, respectively). FDR correction with $\alpha$= 0.05 yields threshold of p < 0.0039.
