# Supplementary material for: Data-Driven Metabolic Pathway Compositions Enhance Cancer Survival Prediction
Source: PLoS Comput Biol. 2016 Sep 27;12(9):e1005125. doi: 10.1371/journal.pcbi.1005125 (PMC5038951; doi:10.1371/journal.pcbi.1005125)
Supplement: S1 Table — (DOCX) [file pcbi.1005125.s004.docx]

**S1 Table** – The AUC and average accuracy for SVM and MCF classifiers for each dataset and the accuracy corresponding paired sample t-test p-value for a 5-fold cross validation procedure.
