# Supplementary material for: Data-Driven Metabolic Pathway Compositions Enhance Cancer Survival Prediction
Source: PLoS Comput Biol. 2016 Sep 27;12(9):e1005125. doi: 10.1371/journal.pcbi.1005125 (PMC5038951; doi:10.1371/journal.pcbi.1005125)
Supplement: S2 Table — (DOCX) [file pcbi.1005125.s005.docx]

**S2 Table** – The AUC and accuracy for the combined datasets of when using SVM vs. MCF for each cancer type and the accuracy corresponding paired sampled p-values for t-test of the 5-fold cross validation.
