# Supplementary material for: Data-Driven Metabolic Pathway Compositions Enhance Cancer Survival Prediction
Source: PLoS Comput Biol. 2016 Sep 27;12(9):e1005125. doi: 10.1371/journal.pcbi.1005125 (PMC5038951; doi:10.1371/journal.pcbi.1005125)
Supplement: S4 Table — (DOCX) [file pcbi.1005125.s007.docx]

**S4 Table** – The frequency of which each breast cancer identified source metabolite was selected over 10,000 repetitions of randomly labeling the datasets and identifying source metabolites that are differentially consumed between cancer and control
