# Supplementary material for: Data-Driven Metabolic Pathway Compositions Enhance Cancer Survival Prediction
Source: PLoS Comput Biol. 2016 Sep 27;12(9):e1005125. doi: 10.1371/journal.pcbi.1005125 (PMC5038951; doi:10.1371/journal.pcbi.1005125)
Supplement: S5 Table — (DOCX) [file pcbi.1005125.s008.docx]

**S5 Table** – The Spearman correlation coefficient (RHO) of (1) the vector of p-values of differential distances vectors from ATP to all other metabolites between cancer and control for the original gene expression datasets and (2) The same vector of p-values for the noisy gene expression datasets for increasing variances.
